# Supplementary material for: Hybrid Orthorhombic Carbon Flakes Intercalated with Bimetallic Au-Ag Nanoclusters: Influence of Synthesis Parameters on Optical Properties
Source: Nanomaterials (Basel). 2020 Jul 15;10(7):1376. doi: 10.3390/nano10071376 (PMC7407132; doi:10.3390/nano10071376)
Supplement: Supplementary file 1 [file nanomaterials-10-01376-s001.pdf]

nanomaterials

Supplementary Material

Hybrid Orthorhombic Carbon Flakes Intercalated with Bimetallic Au-Ag Nanoclusters: Influence of Synthesis Parameters on Optical Properties

M. A. Butt [1,2,3], D. Mamonova [4], Y. Petrov [5], A. Proklova [4], I. Kritchenkov [4], A. Manshina [4,*], P. Banzer [1,2,3,*] and G. Leuchs [1,2,3]

1 Max Planck Institute for the Science of Light, 91058 Erlangen, Germany
2 Institute of Optics, Information and Photonics, University Erlangen-Nuremberg, 91058 Erlangen, Germany
3 School of Advanced Optical Technologies, University Erlangen-Nuremberg, 91052 Erlangen, Germany
4 Institute of Chemistry, St. Petersburg State University, St. Petersburg, Russia
5 Faculty of physics, St. Petersburg State University, St. Petersburg, Russia
* Correspondence: peter.banzer@mpl.mpg.de; alina.manshina@spbu.ru

SMC solution preparation

$(AuC_2Ph)_n$ [1], $(AgC_2Ph)_n$ [2], and $(PhC_2Au)_2\{PPh_2(C_6H_4)_3PPh_2\}$ [3] were synthesized according to published procedures. Other reagents and solvents were used as received. Solution 1H and 31P NMR spectra were recorded on Bruker Avance 400 spectrometer. Microanalyses were carried out in the analytical laboratory of University of Eastern Finland (Department of Chemistry).

$[\{Au_{10}Ag_{12}(C_2Ph)_{20}\}Au_3\{PPh_2(C_6H_4)_3PPh_2\}_3][PF_6]_5$ (SMC) was synthesized according to published procedure [4].

$(PhC_2Au)_2\{PPh_2(C_6H_4)_3PPh_2\}$ (50 mg, 0.042 mmol), $(AuC_2Ph)_n$ (29 mg, 0.097 mmol) and $(AgC_2Ph)_n$ (20 mg, 0.096 mmol) were suspended in acetone (12 ml) and a solution of $AgPF_6$ (18 mg, 0.071 mmol) in acetone (3 ml) was added resulting in an orange solution. The reaction mixture was stirred for 20 hours in the absence of light in argon atmosphere. After filtration, the resulting solution was evaporated and the target compound was recrystallized twice by gas-phase diffusion of pentane into its acetone solution (89 mg, 75%). $^{31}P\{^1H\}$ NMR ($(CD_3)_2CO$; δ): 43.5 (s, 6P), −144.8 (sept, 5P, PF_6). ^1H NMR ($(CD_3)_2CO$; δ): $\{PPh_2(C_6H_4)_3PPh_2\}$: 8.42 (s, $–C_6H_4–$, 12H), 8.35 (d, *m*–H, ($–C_6H_4–P$), 12H, $J_{(H–H)}$ = 8.2 Hz), 7.93 (m, *o*–H, (Ph–P), 24H, $J_{(H–H)}$ = 6.7, $J_{(P–H)}$ = 14 Hz), 7.71 (m, *o*–H, ($–C_6H_4–P$), 12H, $J_{(H–H)}$ = 8.2, $J_{(P–H)}$ = 15 Hz), 7.67 (t, *p*–H, (PhP), 12H, $J_{(H–H)}$ = 7.7 Hz), 7.42 (dd, *m*–H, (Ph–P), 24H, $J_{(H–H)}$ = 8.2, 7.7 Hz); $\{Au(C_2Ph)_2\}$ (three sets A:B:C = 1:3:6); {A}: 7.02 (t, *p*–H, 2H, $J_{(H–H)}$ =.4 Hz), 6.85 (d, *o*–H, 4H, $J_{(H–H)}$ = 7.0 Hz), 5.86 (dd, *m*–H, 4H, $J_{(H–H)}$ = 7.0, 7.4 Hz); {B}: 7.27 (t, *p*–H, 6H, $J_{(H–H)}$ = 7.4 Hz), 7.00 (dd, *m*–H, 12H, $J_{(H–H)}$ = 7.4, 8.0 Hz), 6.49 (d, *o*–H, 12H, $J_{(H–H)}$ = 8.0 Hz); {C}: 7.14 (t, *p*–H, 12H, $J_{(H–H)}$ = 7.5 Hz), 6.92 (d, *o*–H, 24H, $J_{(H–H)}$ = 8.1 Hz), 6.55 (dd, *m*–H, 24H, $J_{(H–H)}$ = 7.5, 8.1 Hz). Anal. calcd for $C_{286}H_{196}Ag_{12}Au_{13}F_{30}P_{11}$: C, 40.90; H, 2.35. Found: C, 40.77; H, 2.38.

References

1. Coates, G.E.; Parkin, C., 621. Gold(I) alkynyls and their co-ordination complexes, C. *J. Chem. Soc.* **1962**, doi:10.1039/JR9620003220.
2. Teo, B.K.; Xu, Y.H.; Zhong, B.Y.; He, Y.K.; Chen, H.Y.; Qian, W.; Deng, Y.J.; Zou, Y.H., A Comparative Study of Third-Order Nonlinear Optical Properties of Silver Phenylacetylide and Related Compounds via Ultrafast Optical Kerr Effect Measurements, *Inorg. Chem.* **2001**, doi:10.1021/ic010408c.
3. Koshevoy, I.O.; Koskinen, L.; Haukka, M.; Tunik, S.P.; Serdobintsev, P.Y.; Melnikov, A.S.; Pakkanen, T.A., Self-Assembly of Supramolecular Luminescent AuI–CuI Complexes: "Wrapping" an Au6Cu6 Cluster in a [Au3(diphosphine)3]3+ "Belt", *Angew. Chemie Int. Ed.* **2008**, doi:10.1002/anie.200800452.

4. Koshevoy, I.O.; Karttunen, A.J.; Tunik, S.P.; Haukka, M.; Selivanov, S.I.; Melnikov, A.S.; Serdobintsev, P.Y.; Pakkanen, T.A., Synthesis, Characterization, Photophysical, and Theoretical Studies of Supramolecular Gold(I)−Silver(I) Alkynyl-Phosphine Complexes, *Organometallics* **2009**, doi:10.1021/om8010036.
